# Inflammation, a Double-Edge Sword for Cancer and Other Age-Related Diseases

**DOI:** 10.3389/fimmu.2018.02160

**Published:** 2018-09-27

**Authors:** Subash Chandra Gupta, Ajaikumar B. Kunnumakkara, Sadhna Aggarwal, Bharat B. Aggarwal

**Affiliations:** ^1^Department of Biochemistry, Institute of Science, Banaras Hindu University, Varanasi, India; ^2^Department of Biosciences and Bioengineering, Indian Institute of Technology, Guwahati, India; ^3^Department of Biotechnology, AIl India Institute of Medical Sciences, New Delhi, India; ^4^Inflammation Research Center, San Diego, California, CA, United States

**Keywords:** cancer, chronic disease, cytokine, inflammation, nutraceutical

## Abstract

Increasing evidence from diverse sources during the past several years has indicated that long-term, low level, chronic inflammation mediates several chronic diseases including cancer, arthritis, obesity, diabetes, cardiovascular diseases, and neurological diseases. The inflammatory molecules and transcription factors, adhesion molecules, AP-1, chemokines, C-reactive protein (CRP), cyclooxygenase (COX)-2, interleukins (ILs), 5-lipooxygenase (5-LOX), matrix metalloproteinases (MMPs), nuclear factor (NF)-kB, signal transducer and activator of transcription 3 (STAT3), tumor necrosis factor (TNF), and vascular endothelial growth factor (VEGF) are molecular links between inflammation and chronic diseases. Thus, suppression of inflammatory molecules could be potential strategy for the prevention and therapy of chronic diseases. The currently available drugs against chronic diseases are highly expensive, minimally effective and produce several side effects when taken for long period of time. The focus of this review is to discuss the potential of nutraceuticals derived from “Mother Nature” such as apigenin, catechins, curcumin, ellagic acid, emodin, epigallocatechin gallate, escin, fisetin, flavopiridol, genistein, isoliquiritigenin, kaempferol, mangostin, morin, myricetin, naringenin, resveratrol, silymarin, vitexin, and xanthohumol in suppression of these inflammatory pathways. Thus, these nutraceuticals offer potential in preventing or delaying the onset of chronic diseases. We provide evidence for the potential of these nutraceuticals from pre-clinical and clinical studies.

The term “inflammation” that means “to set on fire” can be both acute and chronic. Although acute inflammation is beneficial, chronic inflammation is a source for several chronic diseases including cancer, diabetes, and obesity (1). The modern science has delineated the molecular basis of inflammation. The inflammatory molecules and transcription factors such as 5-LOX, adhesion molecules, chemokines, COX-2, C-reactive protein, cytokines, MMPs, NF-κB, prostate-specific antigen (PSA), STAT3, TWIST, and vascular endothelial growth factor (VEGF) are known molecular links between inflammation and chronic diseases (Figure 1) (1). The pro-inflammatory transcription factors (NF-κB and STAT3) are the crucial regulators of inflammation (1, 2). For example, more than 500 cancer related genes are known to be regulated by NF-kB (3, 4).

**Figure 1:**
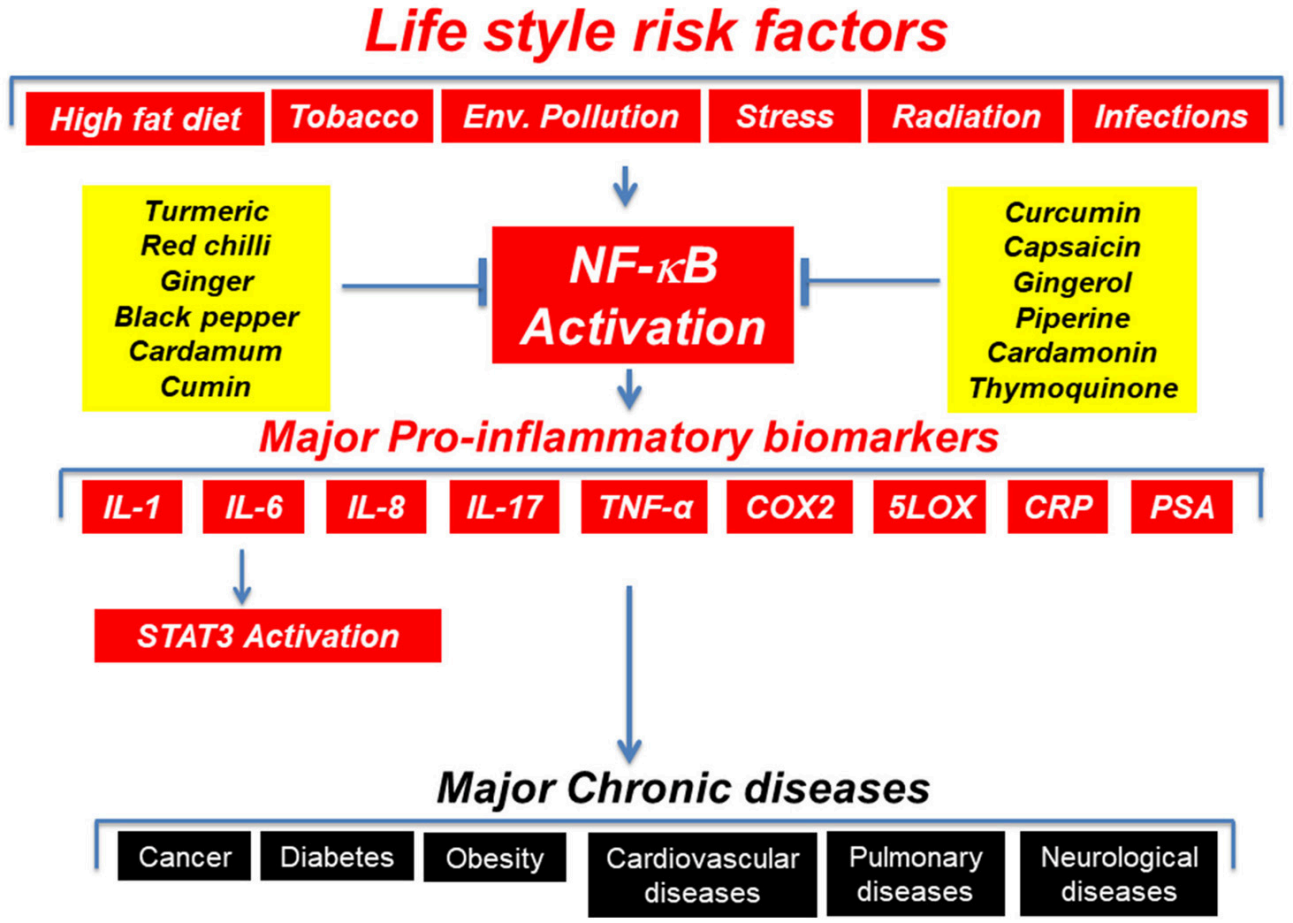
Major risk factors and inflammatory molecules associated with chronic diseases.

The epidemiological, genetic and pharmacological studies support the association of inflammation with chronic diseases (5). For example, accumulating evidence suggest that chronic inflammation is precursor to most tumors. The gastritis (inflammation of lining of stomach) can lead to gastric cancer (6). It is estimated that almost 20% of smokers with bronchitis (inflammation of the mucous membrane in the bronchial tubes) can develop lung cancer in their lifetime (7). Similarly, colitis (inflammation of colon) is a precursor to colon cancer (8). Chronic inflammation plays a crucial role in various aspects of tumor development including cellular transformation, survival, proliferation, invasion, metastasis, and angiogenesis (5, 9). The healthy lifestyle can significantly reduce the risk of developing cancer, cardiovascular diseases, type 2 diabetes, and stroke (10).

The lifestyle factors such as alcohol, infectious agents, obesity, radiation, stress, tobacco, and toxicants are known activators of inflammatory pathways. The dietary intake of low-density lipoproteins can induce inflammation of the arteries. Omega-6 essential fatty acids commonly present in dietary vegetable oils, is known to induce inflammation. However, omega-3 fatty acids can lower inflammation. The dietary dairy protein (casein) and wheat protein (gluten) can also induce inflammation. The environmental sources of inflammation are toxicants such as adhesives, air fresheners, cleaning products, glues, latex, plastics, and synthetic fibers. The inflammation can also be induced by hormonal changes such as estrogen, progesterone, and testosterone. The lifestyle factors are known to modulate the production of inflammatory molecules (11). Lifestyle factors can also induce production of reactive oxygen species (ROS), which in turn lead to inflammation (12–15). ROS can regulate production of several inflammatory molecules such as chemokines, cyclooxygenase-2, cytokines, and pro-inflammatory transcription factors (16).

It is now well known that chronic inflammation is a cause for most chronic diseases. Thus, chronic treatment is required for most chronic diseases. In addition, dysregulation in multiple inflammatory molecules contribute to the development of chronic diseases. Yet, drugs for most of the chronic diseases are based on the modulation of more specifically a single target. Thus, these drugs are less likely to be effective. In addition, these drugs are highly expensive and are associated with numerous side effects when taken for long period of time (17–20). The implication of these facts necessitates the development of agents that are cost-effective, multi-targeted, and readily available. Because of their affordability, safety, and long-term use, agents derived from natural sources (nutraceuticals) possess enormous potential (21, 22). The sources of nutraceuticals include cereals, fruits, nuts, pulses, spices and vegetables. A recent study suggests that more than 70% of the drugs introduced over the past 25 years have been originated from nature (23).

The evidence from pre-clinical and clinical studies support the role of nutraceuticals in suppressing inflammatory pathways. Curcumin, which is derived from the golden spice turmeric, is known to modulate the production as well as activity of a number of inflammatory molecules (24). Curcumin can also directly bind to a number of inflammatory molecules. For example, the molecular docking studies have revealed that curcumin can bind at the receptor-binding sites of TNF-α by forming both noncovalent and covalent interactions (25). Curcumin can also directly bind and inhibit the activities of COX-1, COX-2, and MMP (26, 27). The potential anticancer activities of nutraceuticals by modulating NF-kB activation pathway has been documented by numerous lines of evidence. The nutraceuticals are known to suppress NF-κB activity by modulating several steps such as IKK activation, phosphorylation and degradation of IκBa, p65 nuclear translocation, phosphorylation and acetylation of p65, and p65 DNA binding. The most common nutraceuticals known to inhibit NF-κB activation include caffeic acid phenethyl ester (CAPE) (28), capsaicin (29), curcumin (30), emodin (31), epigallocatechin gallate (EGCG) (32, 33), guggulsterone (34), resveratrol (35, 36), and sanguinarine (29). Some nutraceuticals such as guggulsterone (34) and EGCG (33) act by inhibiting IKK activation. Curcumin (34, 37, 38), guggulsterone (34), capsaicin (29, 39), sanguinarine (29), emodin (31), and EGCG (33) are known to prevent phosphorylation and degradation of IκBα, which is a central point in NF-κB activation. Capsaicin (1, 29, 39) and EGCG (33) are known to inhibit nuclear translocation of NF-κB p65. Nutraceuticals can also inhibit the binding of p65 with DNA. For example, in human myeloid leukemia cells, curcumin was found to inhibit p65-DNA binding (30). Caffeic acid phenethyl ester can suppress the direct binding of the p50-p65 complex with DNA (28). In HeLa cells, emodin can oxidize the redox-sensitive site on NF-κB and thereby can prevent NF-κB-DNA binding (40). Plumbagin can inhibit NF-κB-DNA binding in breast cancer cells (41, 42). Nutraceuticals are also known to sensitize cancer cells to the chemotherapeutic agents and to induce apoptosis through modulation of NF-κB activation pathway. The most common nutraceuticals among this category are anacardic acid (43), 1'-acetoxychavicol acetate (44), noscapine (45), evodiamine (46), indirubin (47), thymoquinone (48), isodeoxyelephantopin, and withanolides (49).

Nutraceuticals are also known to inhibit STAT3 activation pathway and to suppress survival of tumor cells. For example, emodin was found to suppress STAT3 activation and to induce apoptosis in human myeloid cells (50). Similarly, suppression of STAT3 activation by capsaicin was found to induce apoptosis in multiple myeloid cells (51). Curcumin can suppress STAT3 activation pathway and tumor growth in an orthotopic murine model of ovarian cancer (52). Similarly, deguelin induced apoptosis in HTLV transformed T cells by inhibiting STAT3 phosphorylation (53). Quercetin can suppress STAT3 tyrosine phosphorylation and angiogenesis (54).

The clinical studies also support the potential of nutraceuticals in suppressing inflammatory pathways and chronic diseases. The safety, pharmacokinetics, and efficacy of nutraceuticals against numerous chronic diseases has been addressed in a number of human clinical trials. For example, EGCG, which is derived from green tea is reported to have potential against several chronic diseases (55). In prostate cancer patients, tea polyphenols are known to suppress serum levels of PSA, VEGF, and hepatocyte growth factor (HGF) (56, 57). The consumption of green tea is reported to reduce the risk of prostate adenocarcinoma (58). Similarly, black tea is known to decrease the levels of inflammatory biomarkers in colon cancer patients (59). The consumption of tea can also reduce the risk of breast cancer (60), gastric cancer (61), and lung cancer (62). Pomegranate, which is rich in isoflavonoid, such as quercetin, kaempferol, and luteolin, has been used for centuries for medicinal purposes (63). The consumption of pomegranate juice is known to significantly increase PSA doubling time in a phase II clinical trial of prostate cancer patients (64). Furthermore, pomegranate juice can decrease cell proliferation and induce apoptosis (64). The incidence of colorectal, prostate, and lung cancer can be reduced by selenium supplementation (65). The nutraceuticals have shown promise for several other chronic diseases such as acquired immunodeficiency syndrome, acute coronary syndrome, arthritis, atherosclerosis, biliary dyskinesia, cardiovascular disease, cholecystitis, chronic bacterial prostatitis, Crohn's disease, Dejerine-Sottas disease, diabetes, diabetic microangiopathy, diabetic nephropathy, gastric inflammation, gastric ulcer, idiopathic orbital inflammatory pseudotumor, irritable bowel disease, lupus nephritis, oral lichen planus, peptic ulcer, renal conditions, tropical pancreatitis, ulcerative colitis, ulcerative proctitis, uveitis, vitiligo, psoriasis, and β-thalassemia (66). In clinical trials, nutraceuticals have been used as an individual agent and also in combination with other agents. The formulations of nutraceuticals such as capsules, emulsions, liposomes, nanoparticles, powder, and tablets have been used for clinical trials.

In addition to cancer, nutraceuticals are also known to produce beneficial effects in other disease models. For example, an oral administration of curcumin at 375 mg (three times a day for 2 weeks) produced beneficial effects in patients with uveitis (67). Curcumin is also effective in patients with rheumatoid arthritis as demonstrated in clinical trials (68, 69). A short-term, double-blind, crossover study examined the efficacy of this polyphenol in 18 rheumatoid arthritis patients (68). The efficacy of curcumin was also compared with that of phenylbutazone, which is a prescription drug. The patients were administered with phenylbutazone (0.3 g/d) or curcumin (1.2 g/d) for 2 weeks. The anti-rheumatic activities of curcumin were identical with that of phenylbutazone. Furthermore, the polyphenol was very well tolerated and produced no adverse effects in patients. The polyphenol also produced anti-rheumatic activities when combined with diclofenac sodium (69). Additionally, curcumin is known to produce symptomatic relief in patients with peptic ulcers (70). One study examined the potential of curcumin against vitiligo, which is characterized by white patches over the skin on the different body parts (71). A statistically significant repigmentation was observed after 8–12 weeks of curcumin treatment. The polyphenol is known to exhibit anti-psoriatic activity possibly through modulation of phosphorylase kinase (PhK) activity (72). The efficacy of curcumin in Alzheimer's disease patients was examined in a randomized, double-blind, placebo-controlled study (73). The patients were administered with the polyphenol at 1 or 4 g doses. Although curcumin was unable to improve mental status and the serum Aβ40 levels, vitamin E level was increased in patients without any adverse effects (73). The polyphenol also reduces total cholesterol and LDL cholesterol, and increases HDL cholesterol in patients with acute coronary syndrome (74). Overall, these results suggest the beneficial effects of curcumin in patients with acute coronary syndrome. When the polyphenol was administered to 10 healthy volunteers for 7 days, reduction in serum lipid peroxides and total serum cholesterol levels, and an increase in HDL cholesterol was observed (75). In one study, the potential of curcuminoids (NCB-02) in 72 patients with type 2 diabetes (T2DM) was examined (76). The patients were randomized to receive atorvastatin (10 mg, once a day), NCB-02 (300 mg of curcumin, twice a day), or placebo for 8 weeks. The administration of curcumin was associated with an improvement in endothelial function and reduction in oxidative stress (MDA) and inflammatory markers (endothelin-1, IL-6, TNFα) suggesting the potential of curcuminoids against T2DM. However, larger, randomized clinical trials are required to confirm these observations. Like curcumin, resveratrol is also beneficial in T2DM patients (77). More specifically, administration of resveratrol at 1 g/day for 45 days suppressed fasting blood glucose, haemoglobinA1c (HbA1c), insulin and insulin resistance. Furthermore, a significant rise in high density lipoprotein cholesterol was observed after resveratrol treatment (77). In patients with non-alcoholic fatty liver disease (NAFLD), resveratrol significantly reduces the levels of glucose, cholesterol, and liver enzymes ALT and aspartate aminotransferase (78). Resveratrol also decreases the levels of ALT and hepatic steatosis in NAFLD patients (79). Conversely, resveratrol was unable to produce beneficial effects in another clinical trial of NAFLD patients (80). The post-menopausal women are at increased risk of breast cancer owing to reduced expression of sex steroid hormone binding globulin (SHBG). Furthermore, a lower ratio of 2-hydroxyestrone (2-OHE1) and 16α-hydroxyestrone (16α-OHE1) in postmenopausal cohort correlate with the higher breast cancer risk (81). An administration of resveratrol at 1 g/day for 12 weeks is known to increase SHBG levels in obese postmenopausal women (82). Resveratrol also elevates 2-OHE1/16α OHE1 ratio. Thus, it can be concluded that resveratrol has beneficial effects in postmenopausal women (82).

In conclusion, chronic inflammation is a cause for several chronic diseases. Thus, treatment of chronic diseases requires chronic treatment. Modern science has delineated the molecular links of chronic inflammation and chronic diseases. The drugs developed by pharmaceutical companies are highly expensive, produce side effects and cannot be afforded by more than 80% of world population. Nutraceuticals have also been successfully used in combination with other agents. Nutraceuticals are readily available and can modulate multiple cell signaling pathways. In addition, nutraceuticals and their sources have been consumed since ancient time. Thus, their safety is well tested. Conversely, nutraceuticals have been reported to produce undesired adverse effects by some studies. For example, oral intake of curcumin is associated with diarrhea, headache, rash, and yellow stool in some healthy volunteers. When curcumin was administered in combination with gemcitabine, abdominal pain was reported by some pancreatic cancer patients. Furthermore, nutraceuticals such as curcumin and resveratrol are associated with poor bioavailability. Overall, nutraceuticals offer promise to prevent or delay the onset of chronic diseases. However, none of the nutraceuticals have been approved for human use by regulatory entities. Moreover, nutraceuticals have been reported to produce adverse effects by some studies. More studies are required before these agents can be prescribed by clinicians for therapeutic purpose.

## Author contributions

All authors listed have made a substantial, direct and intellectual contribution to the work, and approved it for publication.

### Conflict of interest statement

The authors declare that the research was conducted in the absence of any commercial or financial relationships that could be construed as a potential conflict of interest.
